# *LSCHL4* from *Japonica* Cultivar, Which Is Allelic to *NAL1*, Increases Yield of *Indica* Super Rice 93-11

**DOI:** 10.1093/mp/ssu055

**Published:** 2014-05-02

**Authors:** Guang-Heng Zhang, Shu-Yu Li, Li Wang, Wei-Jun Ye, Da-Li Zeng, Yu-Chun Rao, You-Lin Peng, Jiang Hu, Yao-Long Yang, Jie Xu, De-Yong Ren, Zhen-Yu Gao, Li Zhu, Guo-Jun Dong, Xing-Ming Hu, Mei-Xian Yan, Long-Biao Guo, Chuan-You Li, Qian Qian

**Affiliations:** ^a^State Key Laboratory of Rice Biology, China National Rice Research Institute, 359 Tiyuchang Road, Hangzhou 310006, Zhejiang, China; ^b^State Key Laboratory of Plant Genomics, National Center for Plant Gene Research, Institute of Genetics and Developmental Biology, Chinese Academy of Sciences, Beijing 100101, China

**Keywords:** rice breeding, QTL, *qLSCHL4*, panicle type, pleiotropism, yield potential.

## Abstract

The basic premise of high yield in rice is to improve leaf photosynthetic efficiency and coordinate the source–sink relationship in rice plants. Quantitative trait loci (QTLs) related to morphological traits and chlorophyll content of rice leaves were detected at the stages of heading to maturity, and a major QTL (*qLSCHL4*) related to flag leaf shape and chlorophyll content was detected at both stages in recombinant inbred lines constructed using the *indica* rice cultivar 93-11 and the *japonica* rice cultivar Nipponbare. Map-based cloning and expression analysis showed that *LSCHL4* is allelic to *NAL1*, a gene previously reported in narrow leaf mutant of rice. Overexpression lines transformed with vector carrying *LSCHL4* from Nipponbare and a near-isogenic line of 93-11 (*NIL-*9311) had significantly increased leaf chlorophyll content, enlarged flag leaf size, and improved panicle type. The average yield of *NIL*-9311 was 18.70% higher than that of 93-11. These results indicate that *LSCHL4* had a pleiotropic function. Exploring and pyramiding more high-yield alleles resembling *LSCHL4* for super rice breeding provides an effective way to achieve new breakthroughs in raising rice yield and generate new ideas for solving the problem of global food safety.

## SUMMARY

The basic premise of high yield in rice is to improve leaf photosynthetic efficiency, and coordinate the source–sink relationship in rice plants. The quantitative trait loci (QTLs) *qLSCHL4, japonica NAL1* allele from Nipponbare has a pleiotropic function, effectively increased leaf chlorophyll content, enlarged flag leaf size, and enhanced the yield of *indica* rice cultivar.

## INTRODUCTION

Rice is a major food crop worldwide, especially in developing countries (Pan et al., 2013; Takai et al., 2013). According to the estimation of global population growth, total rice production of 2001–30 should maintain an annual growth rate of 1.2% in order to meet the future demand for food (Yuan, 2004; van Nguyen and Ferrero, 2006). Over the past decade, breakthroughs in raising the yield potential in rice through conventional breeding have been limited, while stagnant and declining yields of rice per unit area have aroused great attention from the global community (Peng et al., 2008, 2009). With global population growth, the contradiction of food supply and demand becomes increasingly prominent. How to achieve breakthroughs in raising the yield of super rice cultivars becomes a new challenge encountered by scientists in solving the issues of global food safety.

The yield of rice is mainly determined by the relative scale and coordination level of source–sink flow (Wang et al., 2005). In selection breeding, molecular markers become extremely popular among breeders and scientists (Li et al., 2012). Using molecular biology techniques to regulate leaf morphological traits and improve yield-related traits (e.g. seed-setting rate, the number of spikelets per panicle, and the number of primary and secondary branches per panicle) is presently the major means for breakthroughs in raising rice yield (Khush, 1990; Zhu et al., 1997). In China, significant progress is achieved in gene exploration and genetic studies of yield-related traits in rice; a number of quantitative trait loci (QTLs) of important traits in rice are detected in different genetic populations (Wang et al., 2011; Marathi et al., 2012; Wang et al., 2012). To date, nearly 20 yield-related QTLs have been isolated by scholars using map-based cloning methods, such as *Gn1a*, *Ghd7*, *DEP1*, *NAL1* (*SPIKE* and *GPS*), and *IPA1*; these genes control plant architecture and panicle type, ultimately affecting the yield of rice mainly through pleiotropic effects (Ashikari et al., 2005; Xue et al., 2008; Huang et al., 2009; Jiao et al., 2010; Fujita et al., 2012, 2013).

*Grain number 1a* (*Gn1a*) encodes cytokinin oxidase (OsCKX2). *Gn1a* mutation inhibits *OsCKX2* gene expression in the inflorescence meristem and causes cytokinin accumulation, thus increasing grain number and improving rice yield. After molecular modification, a near-isogenic line carrying the *sd1* allele in the Koshihikari background (NIL-*sd1*) has decreased grain number and plant height, whereas NIL-*Gn1a* gains increased grain number by 34% and NIL-*sd1+Gn1a* obtains increased grain number by 23% but decreased plant height compared with Koshihikari. This provides a new strategy for improving crop yield (Ashikari et al., 2005). In the rice cultivar Minghui 63, *Ghd7* allele delays the heading stage while significantly increasing plant height and the number of spikelets per panicle (Xue et al., 2008; Guo et al., 2013); additionally, this allele increases stem diameter, enhances lodging resistance, and improves the yield per unit area by up to 50% (Xue et al., 2008). *Dense and erect panicle 1* (*DEP1*) is a regulatory gene of panicle type in rice, which encodes a protein containing PEBP-like domain and promotes cell division. *DEP1* multination is associated with high-yield traits such as shortening of stem and increases in the seed-setting density, the number of primary and secondary branches per panicle, and the number of spikelets per panicle, ultimately increasing rice yield by 15%–20% (Huang et al., 2009). *Ideal Plant Architecture 1* (*IPA1*) is a major QTL that controls the ideal plant type of rice and encodes an SBP-box transcription factor regulated by microRNA OsmiR156 for translation and stability. Rice plants carrying *IPA1* feature declines in number of tillers, and increases in stem diameter, the number of spikelets per panicle, and thousand-grain weight, which contribute to the yield increase by approximately 10% (Jiao et al., 2010). *Narrow leaf 1* (*NAL1*), a gene that regulates the development of vascular bundle, was first cloned from loss-of-function narrow leaf mutant in rice by Qi et al. (2008). Previously, authors consider that *NAL1* plays its role in leaf morphogenesis by regulating polar auxin transport, and plays a regulatory role in the development of plant type in rice (Chen et al., 2012; Fujita et al., 2013; Takai et al., 2013).

In the present study, we constructed recombinant inbred lines (RILs) of the super rice cultivar 93-11 (*Oryza sativa* L. ssp. *indica* ‘Yangdao 6’) and the popular high-quality *japonica* cultivar Nipponbare (*Oryza sativa* L. ssp. *japonica*). A major QTL (*qLSCHL4*) that regulates leaf morphology and chlorophyll content was isolated using map-based cloning technique. Functional validation of *LSCHL4* was accomplished based on the construction of overexpression vectors, combined with the development of linked molecular markers. To further clarify the impact of *LSCHL4* on rice yield, we conducted a field plot experiment with 93-11 and a near-isogenic line (NIL-9311) in the genetic background of *indica*. The results demonstrate that transformation of *japonica*-derived gene *LSCHL4* into rice cultivars with *indica* as the genetic background effectively increases the numbers of secondary branches and grains per panicle, thereby significantly increasing the rice yield. It is recommend pyramiding more high-yield alleles from *japonica* for super rice breeding with *indica* background by molecular design, in order to achieve new breakthrough in raising the yield in super rice, and further to provide new ideas for solving the problem of global food safety.

## RESULTS

### Mapping of QTLs for Leaf Morphological Traits and Soil Plant Analyzer Development (SPAD) Value

**Figure 1:**
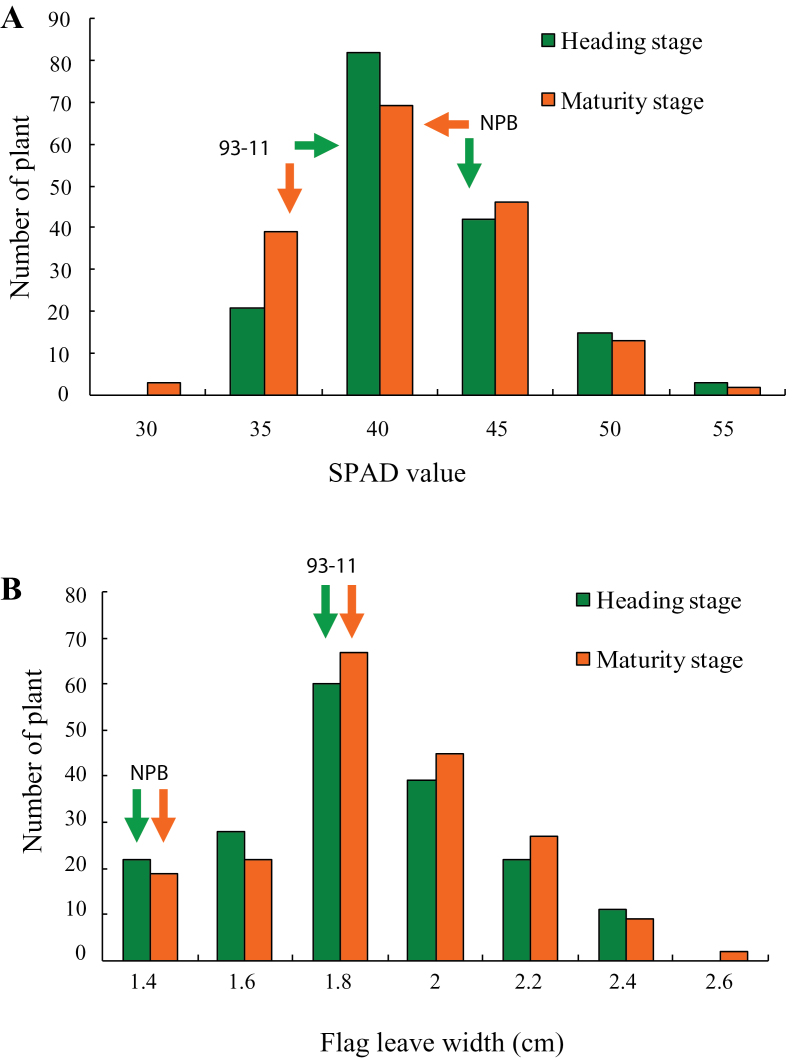
Frequency Distribution of SPAD Value (A) and Flag Leaf Width (B) in RILs Population Constructed by *O. sativa* ssp. *indica cv.* 93-11 and *O. sativa* ssp. *japonica cv.* Nipponbare at Heading Stage (Green Boxes) and Maturity Stage (Orange Boxes). **(A)** Arrows indicate the SPAD score of parents 93-11 and Nipponbare at heading stage (green) and maturity stage (orange), respectively. **(B)** Arrows indicate flag leaf width of parents 93-11 and Nipponbare at heading stage (green) and maturity stage (orange), respectively.

Leaf morphology and chlorophyll content in rice undergo certain changes at various growth stages (Jiang et al., 2012). In the present study, we determined flag leaf width and Soil Plant Analyzer Development (SPAD) value (a parameter that could measure the relative content of chlorophyll or be on behalf of the plant green degree) in the parental cultivars and 207 segregating individuals of a RIL population. Both the above traits had significant differences between the two parental cultivars and displayed continuous distribution among the segregating individuals. For both traits, there existed transgressive segregation in the RIL population (Figure 1), and thus were suitable for interval mapping.

**Figure 2:**
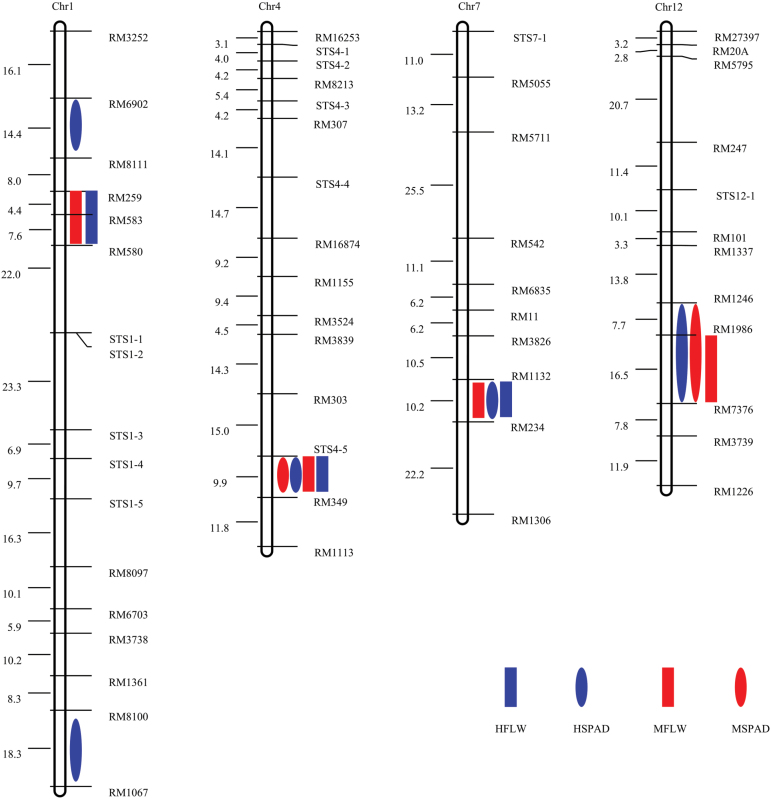
Genetic Linkage Map of Putative QTLs for Flag Leaf Width and Cholorphyll Content at Different Growth Stages in Rice. Chromosome numbers are indicated above each linkage map. Numbers to the left of the linkage map represent interval genetic distance (cm), and marker names are to the right. *Ovals* and *boxes* to the right of the linkage map represent LOD peaks of putative QTLs. HFLW, heading stage flag leaf width (blue box); HSPAD, heading stage SPAD score (blue oval); MFLW, maturity stage flag leaf width (red box); MSPAD, maturity stage SPAD score (red oval).

At heading and maturity, two different growing stages, a total of 14 QTLs were detected in six different fragments on four chromosomes (Table 1 and Figure 2). These included three QTLs for leaf width and five QTLs for SPAD value at the heading stage, and four QTLs for leaf width and two QTLs for SPAD value at the maturity stage. The QTLs detected for leaf width had no significantly difference at the two stages, indicating that flag leaf morphology had generally stabilized at the heading stage; because leaf width had no substantial differences compared with those at the maturity stage, relevant QTLs were very likely detected at different stages repetitively. This situation was different for leaf chlorophyll content, and the number of QTLs for SPAD value declined by 50% from the heading to maturity stage, possibly due to reduced stay-green trait and fast aging rate at a late growth stage of *indica* rice cultivar relative to *japonica* rice cultivar. In total, four QTL intervals for leaf width were detected at the heading and maturity stages, three of which were detectable in both periods (RM259-RM580 on chromosome 1, STS4-5-RM349 on chromosome 4, and RM1132-RM234 on chromosome 7). In addition, the QTLs of *qFLW-1* and *qFLW-7*, the other QTLs associated with flag leave width additive effect contributed alleles were from the parental cultivar Nipponbare. Similarly, there were two QTL intervals for SPAD value detected at both growth stages (STS4-5-RM349 on chromosome 4 and RM1246-RM7376 on chromosome 12, respectively), but their additive effect alleles came from an opposite direction.

**Table 1:** Flag Leaf Width and SPAD Score QTL Detected in the 93-11 × Nipponbare RIL Population during Heading Stage and Maturity Stage

Growing stage	Trait	QTL	Ch.	Interval		LOD score	Additive effect	*R* ^2^ (%)
Heading stage	Leaf width	HFLW-1	1	RM259	RM580	5.40	0.48	7.2
		HFLW-4	4	STS4-5	RM349	7.84	–0.97	14.8
		HFLW-7	7	RM1132	RM234	6.50	0.68	12.3
	SPAD value	HSPAD-1a	1	RM8100	RM1067	4.25	–1.90	11.3
		HSPAD-1b	1	RM6902	RM259	3.75	–2.36	8.1
		HSPAD-4	4	STS4-5	RM349	9.24	–3.84	20.8
		HSPAD-7	7	RM1132	RM234	4.28	–2.49	13.9
		HSPAD-12	12	RM1246	RM7376	3.41	2.43	10.6
Maturity stage	Leaf width	MFLW-1	1	RM259	RM580	4.1	0.56	8.4
		MFLW-4	4	STS4-5	RM349	6.94	–1.10	12.1
		MFLW-7	7	RM1132	RM234	4.40	1.05	9.4
		MFLW-12	12	RM1986	RM7376	2.83	–0.83	7.7
	SPAD value	MSPAD-4	4	STS4-5	RM349	6.46	–2.31	13.5
		MSPAD-12	12	RM1246	RM7376	3.49	1.70	8.7

HFLW, heading stage flag leaf width; HSPAD, heading stage SPAD value; MFLW, maturity stage flag leaf width; MSPAD, maturity stage SPAD value. Positive and negative values indicate additive effects contributed by the alleles of 93-11 and Nipponbare, respectively.

The additive effect allele *qSPAD-4* (including *HSPAD-4* and *MSPAD-4*) located on chromosome 4 was from the *japonica* rice cultivar Nipponbare, which increased SPAD value by 3.84 at the heading stage and accounted for 20.8% of genetic variation; this allele increased SPAD value by 2.31 at the maturity stage and accounted for 13.5% of genetic variation. The additive effect allele *qSPAD-12* (including *HSPAD-12* and *MSPAD-12*) located on chromosome 12 was from 93-11, which increased the trait value by 2.43 at the heading stage and by 1.70 at the maturity stage, accounting for 10.6% and 8.7% of genetic variation, respectively.

Regardless of the growth stage of heading or maturity, a QTL related to both leaf width and SPAD value was detected at the STS4-5-RM349 interval on the long arm of chromosome 4, and both additive effect alleles came from Nipponbare. These results indicate that the STS4-5-RM349 interval on chromosome 4 may contain important gene(s) that regulates the development of leaf morphological traits and chlorophyll synthesis in rice, further playing a regulatory role in plant growth and development. Whether there exists a pleiotropic gene or closely linked multi-genes needs to be further verified.

### Leaf Chlorophyll Content and Morphological Traits in Near-Isogenic Line NIL-9311

**Figure 3:**
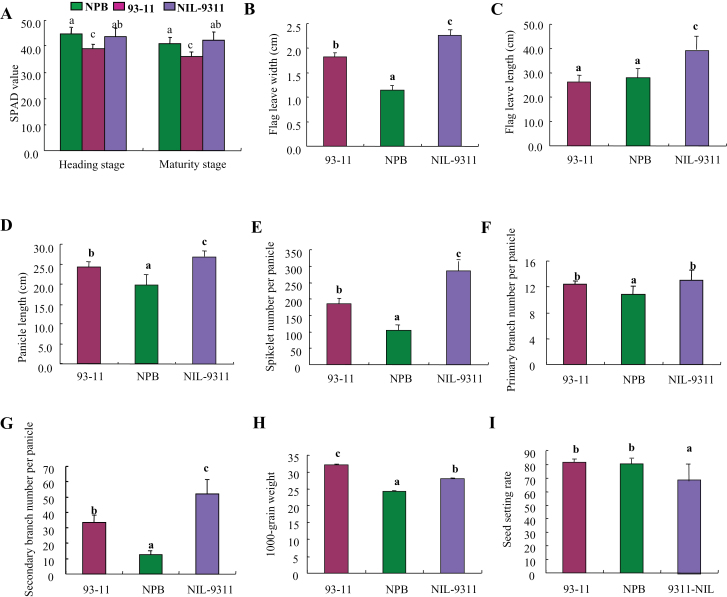
Characterization of Flag Leave and Yield-Related Traits of 93-11, NPB, and NIL-9311. **(A)** SPAD value of flag leaves at heading stage and maturity stage (20 d after heading stage). **(B–I)** Comparison of flag leave width (B) and length (C), primary panicle length (D), the spikelet number (E), primary branch number (F), and secondary branch number (G) per panicle, 1000-grain weight (H), and seed-setting rate (I). Each column represents mean ± SD. Columns with different letters were significantly different (*P* < 0.05, least significant difference test).

Chlorophyll is the pigment for plant photosynthesis in rice leaves, which, together with leaf morphological traits, jointly affects leaf photosynthetic efficiency and directly affects the yield of rice (Zhang et al., 2009). In the present study, we surveyed SPAD value of flag leaves in the near-isogenic line NIL-9311 at the initial heading and maturity stages (20 d after full heading), as well as yield-related leaf morphological traits and panicle traits at the maturity stage. Results showed that at the initial heading stage, average SPAD values of flag leaves were 44.8 in Nipponbare and 38.9 in 93-11; compared with the trait value of 93-11, average SPAD value of flag leaves in NIL-9311 increased by 12.08%, namely by 43.6. At the maturity stage, the SPAD values of flag leaves significantly declined in the parents and near-isogenic line (41.2 and 36.2 versus 42.1, respectively) compared with the trait values at the full heading stage; however, the trait value of NIL-9311 remained 16.30% greater than that of 93-11 (Figure 3A). The above results demonstrated that, regardless of the growth stages of heading or maturity, the near-isogenic line NIL-9311 containing a chromosomal fragment from the *japonica* rice cultivar Nipponbare gained significantly higher chlorophyll content than the parental cultivar 93-11 within the genetic background of *indica*. Thus, the *japonica* chromosomal fragment may contain important gene(s) capable of increasing leaf chlorophyll content in 93-11, further playing a role in the stay-green of leaves and the delay of aging at late growth stages of rice.

In addition, we conducted a comparative analysis on leaf morphological traits in both the parental cultivars (93-11 and Nipponbare) and NIL-9311. Results showed that 93-11 and Nipponbare had a highly significant difference in flag leaf width (1.82 versus 1.24cm), with an average gap of 0.58cm. And NIL-9311 had significantly greater flag leaf width than the parents; average flag leaf width of NIL-9311 was 2.19cm, which increased by 20.33% and 70.96% compared to the traits values of donor (93-11) and receptor parents (Nipponbare), respectively (Figure 3B). These results are consistent with our findings that the QTL for leaf width originates from Nipponbare. The increase in leaf width of NIL-9311 mainly resulted from the substitution of chromosomal fragments containing the QTL *LSCHL4* which were detected at the heading and maturity stages in the same QTL interval STS4-5-RM349 on chromosome 4, including *HFLW-4*, *MFLW-12*, *HSPAD-4*, and *MSPAD-4* from Nipponbare, as there is a major gene in this gene fragment capable of increasing flag leaf width in rice.

Regarding flag leaf length, there was no significant difference between the donor and receptor parents. In contrast to the trait values of leaf width, flag leaf length was 1.68cm greater in Nipponbare than in 93-11 (27.98 versus 26.3cm). Compared with the trait values of both parental cultivars, the near-isogenic line 9311-*LSCHL4* had highly significantly increased flag leaf length, similar to the increasing trend in flag leaf width. Flag leaf length averaged 34.58cm in the NIL-9311, which increased by 31.48% and 23.59% compared to 93-11 and Nipponbare, respectively (Figure 3C). Combined with the above results, we consider that there exists a major gene(s) in the STS4-5-RM349 interval related to both flag leaf morphological development chlorophyll synthesis. Whether it is a pleiotropic gene or two closely linked genes remains unknown.

### Yield-Related Traits in NIL-9311

Panicle traits and grain traits are important indicators for assessing the yield in rice, which eventually determines the crop yield. In this study, we surveyed panicle traits (panicle length, the number of spikelets per panicle, seed-setting rate, and the numbers of primary and secondary branches per panicle) in both the parental cultivars and NIL-9311 carrying *LSCHL4* from Nipponbare. Results showed that the parents and NIL-9311 had significantly different panicle traits. Compared with the recurrent parent 93-11, the NIL-9311 had significant increases in panicle length, the number of spikelets, and the number of secondary branch per panicle; there was no significant change in the number of primary branch per panicle, with declines in seed-setting rate and thousand-grain weight to different extents (Figure 3D–3I). These results indicate that the major gene present in *LSCHL4* containing chromosomal fragment from Nipponbare plays an important regulatory role in the development of panicle morphology and grain traits.

### Leaf Morphology and Tissue Structure in 93-11 and NIL-9311

**Figure 4:**
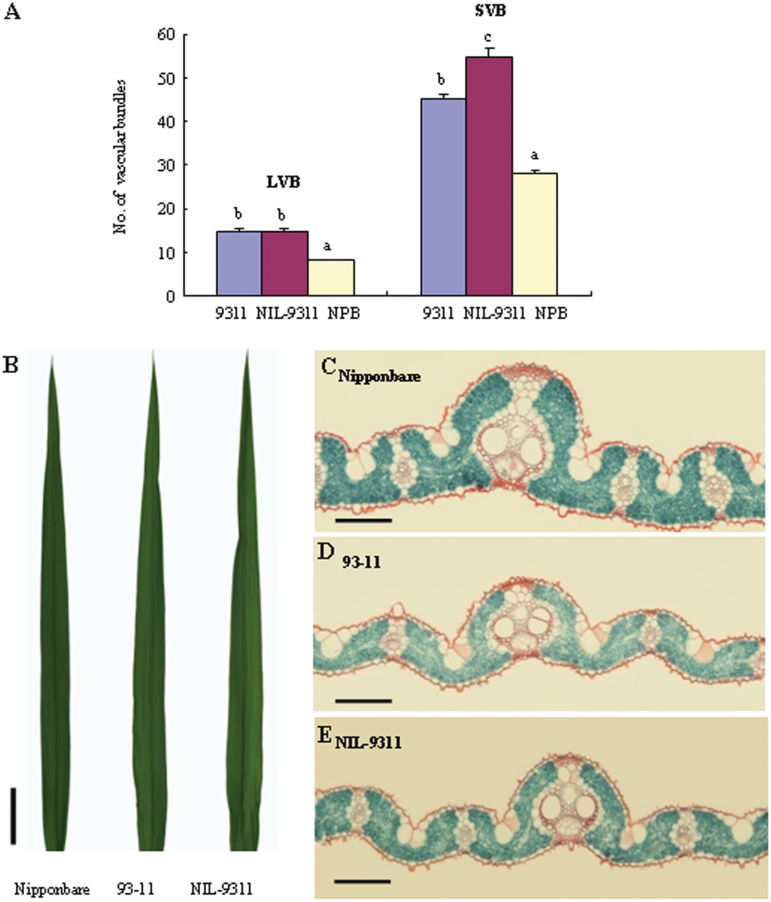
Comparison Morphology and Cross-Sections of Flag Leaves. **(A)** Characterization of large vascular bundle (LVB) and small vascular bundle (SVB) number of 93-11, NIL-9311, and NPB. Each column represents mean ± SD. Columns with different letters were significantly different (*P* < 0.01, least significant difference test). **(B)** Flag leave morphology of Nipponbare, 93-11, and NIL-9311 (bar = 2.5 cm). **(C–E)** Cross-sections of flag leaves stained with Safranin O and Fastgreen FCF in Nipponbare, 93-11, and NIL-9311. Bar = 100 μm.

The results of histological examination showed that NIL-9311 had significantly increased large and small bundles in flag leaves relative to 93-11 (Figure 4A and 4B). According to statistics, there was no significant difference in the number of large vascular bundles in flag leaves between NIL-9311 and 93-11 (13.81 versus 14.10), whereas the average number of small vascular bundles in flag leaves was significantly greater in the former than in the latter (54.60 versus 44.80), showing an increase of 21.88%.

In the middle cross-section of rice leaf blade, the area occupied by mesophyll cells significantly increased in NIL-9311 carrying the *japonica* allele *LSCHL4* compared to that in 93-11. Increased mesophyll cells per unit area might be the primary reason for chlorophyll increase in NIL-9311. We propose that the *japonica NAL1* allele *LSCHL4* promotes the enlargement of flag leaf area (mainly small bundles and mesophyll cells) in *indica* rice, leading to leaf chlorophyll increase in NIL-9311 (Figure 4B–4E).

### Map-Based Cloning and Identification of qLSCHL4

**Figure 5:**
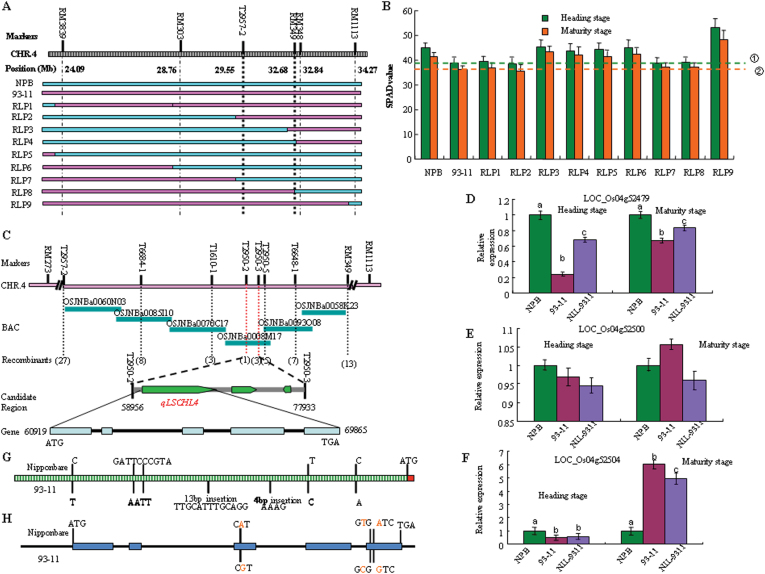
*qLSCHL4* Cloning on Chromosome 4. **(A)** Substitution mapping of QTLs controlling SPAD value of flag leaves at heading stage and maturity stage (20 d after heading stage) on chromosome 4 based on nine RILs. Graphical genotypes. Blue denotes regions homozygous for Nipponbare alleles; pine denotes regions homozygous for 93-11 alleles. SPAD values of flag leaves. **(B)** *Green box* column means heading stage and *orange box* means maturity stage; each column represents mean ± S.D. *line* ① means the SPAD value of flag leaf of 93-11 at heading stage, and *line* ② means the SPAD value of flag leaf of 93-11 at maturity stage. **(C)** Fine mapping of *qLSCHL4* on chromosome 4. *qLSCHL4* was mapped primarily to the long arm of rice chromosome 4 between markers T2957–2 and RM348, and then narrowed to a 18.977-kb region between T2950–2 and T2950–3 on the clone OSJNBa0008M17. **(D–F)** Relative expression of three candidate genes LOC_Os04g52479, LOC_Os04g52500, and LOC_Os04g52504 at heading and maturity stages by qRT–PCR. Columns with different letters were significantly different (*P* < 0.05, least significant difference test). **(G)** Sequence differences sites in the promoter (2 kb) upstream the initiation codon (ATG) of q*SLCHL4* between 93-11 and Nipponbare. **(H)** Red letters means the difference sites in the ORF of q*SLCHL4* between 93-11 and Nipponbare.

Using 1700 individuals of the BC_7_F_2_ population, we preliminarily positioned *LSCHL4* between T2957–2 and RM349. Then, the peripheral molecular markers RM3839 and RM1113 were used to screen out nine different types of RILs for verification of SPAD measurement (Figure 5A and 5B). Results confirmed that there was a major gene related to chlorophyll synthesis between T2957–2 and RM349.

By further expanding the population to 6790 segregating individuals, we finally positioned *LSCHL4* in an 18.879-kb fragment between two STS markers, T2950–2 and T2950–3 (Figure 5C). According to genome annotation databases (http://rice.plantbiology.msu.edu/cgi-bin/gbrowse/rice/#search), the 18.98-kb region contains three common predicted genes: LOC_Os04g52479, LOC_Os04g52500, and LOC_Os04g52504. These genes were predicted to encode the following proteins: peptidase, trypsin-like serine and cysteine proteases, lecithine cholesterol acyltransferase, and adhesive/proline-rich protein.

The cDNA sequences of three candidate genes were predicted (http://rice.plantbiology.msu.edu/cgi-bin/gbrowse/rice/#search) and then expressed in rice at the heading and maturity stages using real-time PCR technique (Figure 5D–5F). Results showed that the relative expression level of LOC_Os04g52500 in rice had no significant changes between the parents at heading and maturity stages. Specifically, 93-11 had a slightly higher expression level of LOC_Os04g52500 at the heading stage but a lower expression level of LOC_Os04g52500 at the maturity stage than Nipponbare, whereas the near-isogenic line NIL-9311 had a consistently lower LOC_Os04g52500 expression level than both parents (Figure 5E).

Compared to LOC_Os04g52500, the remaining two genes, LOC_Os04g52479 and LOC_Os04g52504, were expressed at different levels in rice at the two growth stages. Regardless of the growth stage of heading or maturity, the relative expression level of allele LOC_Os04g52479 was significantly higher in Nipponbare than in 93-11. Specifically, LOC_Os04g52479 expression level in Nipponbare was four-fold greater than that in 93-11 at the heading stage; despite the slight declines at the maturity stage, the gene expression level in Nipponbare was 1.5-fold higher than that in 93-11. The corresponding gene expression level in NIL-9311 was between data of the parents, with a significant increase relative to data of 93-11 (Figure 5D). The above results indicate that the expression of allele LOC_Os04g52479 derived from Nipponbare significantly increases in rice with the genetic background of 93-11.

The third candidate gene, LOC_Os04g52504, was expressed at low levels in both parents at the heading stage, one-fold higher in Nipponbare than in 93-11; the corresponding gene expression in NIL-9311 was significantly higher than that in 93-11. At the maturity stage, however, the LOC_Os04g52504 expression level in 93-11 increased significant, nearly six-fold higher than that in Nipponbare; the corresponding gene expression level in NIL-9311 became lower than that in 93-11 and approximately four-fold higher than that in Nipponbare (Figure 5F). According to the above results, we inferred that LOC_Os04g52479, tentatively named *SLCHL4*, is a major gene related to both chlorophyll synthesis and leaf morphological development. *SLCHL4* has a consistent sequence with previously reported *NAL1* that controls the development of vascular bundles via auxin transport.

### Allelic Sequence Comparison of SLCHL4 in 93-11 and Nipponbare

According to the predicted data, we sequenced the promoter (2kb before ATG) and genome of *SLCHL4* in 93-11 and Nipponbare. Results showed that the two parents had base differences at seven positions in the promoter region, including three single-base substitutions and four multi-base insertions and deletions (Figure 5G); in the coding region, there were one and two single-base substitutions on the third and fifth exons, respectively, causing the changes in the amino acids encoded (Figure 5H). We speculated that the difference in promoter sequence and the changes in amino acid coding region led to differences in the structure and expression level of protein encoded by this gene.

### Impacts of LSCHL4 Overexpression on Yield-Related Traits in Japonicas

**Figure 6:**
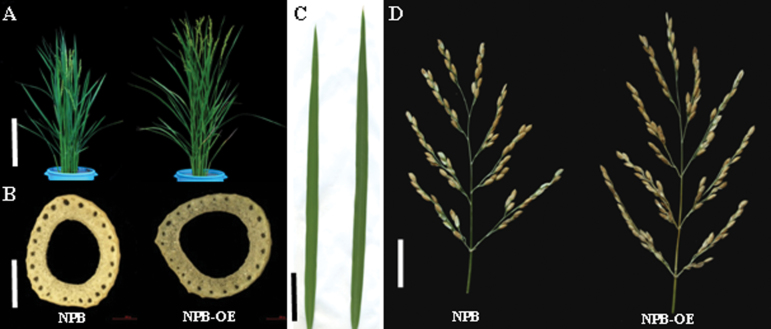
Morphologies of Nipponbare (NPB) Plant and Overexpressor (Ubi:qLSCHL4) Transgenic Nipponbare Plant (NPB-OE). **(A)** Plant morphology (bar = 30 cm). **(B)** The culm cross-section of the fourth internode (bar = 2 mm). **(C)** Leaf shape (bar = 5 cm). **(D)** Panicle (bar = 4 cm) of Nipponbare (NPB) and overexpression Niponbare (NPB-OE).

**Figure 7:**
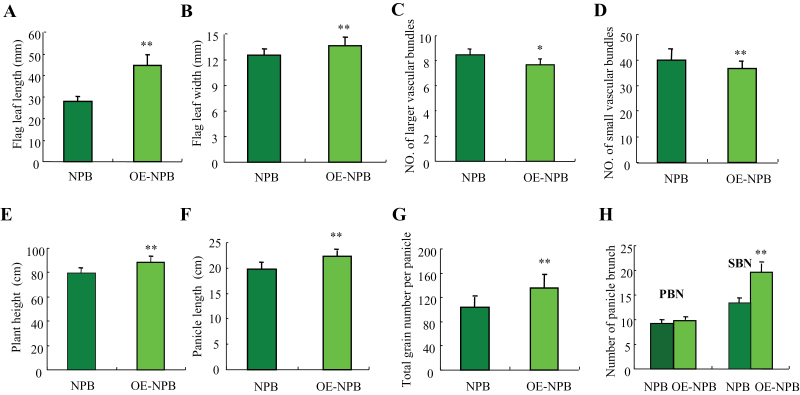
Transgenic Analysis for *qLSCHL4* through Overexpression. Comparison of flag leave length **(A)** and width **(B)**; number of large **(C)** and small **(D)** vascular bundles; plant height **(E)**, panicle length **(F)**, total grain number per panicle **(G)**, and number of panicle brunch **(H)**. PBN, primary branch number per panicle; SBN, secondary branch number per panicle. Each column represents mean ± SD (*n* = 10). * and ** indicate significant differences compared NIL-9311 to Nipponbare at 0.05 and 0.01 levels, respectively.

To determine whether the *LSCHL4* gene affects yield-related traits in rice, we constructed an overexpression vector for cDNA of *LSCHL4* from Nipponbare. This vector was transformed into the *japonica* cultivar Nipponbare and an *LSCHL4* overexpression line (OE-NPB) was surveyed in terms of leaf morphology and panicle traits. Results showed that, compared with Nipponbare, OE-NPB had enlarged plant morphological traits (Figure 6). Overexpression of *LSCHL4* from *japonica* increased multiple organs to different extents, including plant height, stem diameter, leaf blade, and panicle size. Despite the increases in flag leaf width and length as well as leaf area, the numbers of both large and small vascular bundles declined in OE-NPB (Figure 7). The increase in leaf width might be caused mainly through the increase in average distance between vascular bundles.

### Increased Yield of NIL-9311 in Field Cultivation

Results showed that NIL-9311 gained higher yield than 93-11 both in theory and in practice. Regarding panicle traits, the thousand-grain weight, seed-setting rate, and number of effective panicles declined in NIL-9311 compared with those in 93-11 (by 8.04%, 3.90%, and 6.91%, respectively). On the contrary, the number of secondary branches and spikelets per panicle increased significantly in NIL-9311 compared with those in 93-11. Specifically, the number of secondary branches per panicle was 48.5 in NIL-9311, which is 14.9 (44.3% increment) greater than that in 93-11; the number of spikelets per panicle was 84.6 larger (46.01% increment) in NIL-9311 compared with that in 93-11 (183.9). Although the seed-setting rate per panicle relatively declined in NIL-9311 compared with that in 93-11, the final number of grains per panicle increased by 49.7 (27.02%) in the former compared with that in the latter.

The measured data of rice yield showed that NIL-9311 gained significant increase in grain yield compared with the parental cultivar 93-11. In the plot of NIL-9311, effective yield was 10.98kg, equivalent to the average yield per hectare of 9152.9kg; in the plot of 93-11, effective yield was 9.25kg, equivalent to the average yield per hectare of 7713.3kg, showing an increasing rate of 18.70% (Table 2). Therefore, we considered that the increase in the grain yield of NIL-9311 was mainly caused by increases in the numbers of secondary branches and grains per panicle. Our results demonstrate that transformation of *japonica*-derived gene *LSCHL4* into rice cultivars with *indica* as the genetic background effectively increases the numbers of secondary branches and grains per panicle, thereby significantly increasing the rice yield.

**Table 2:** Yield Test in a Paddy between 93-11 and NIL-9311

Trait	93-11	NIL-9311
Panicles per plot	2139.58±104.17	1991.67±145.83**
Number of primary branches	12.4±0.5	12.8±0.8
Number of secondary branches	33.6±4.2	48.5±8.6**
Grains per panicle	183.9±15.5	268.5±24.7**
Seed-setting rate	80.53±2.32	77.39±2.17*
1000-grain weight (g)	31.31±0.26	28.79±0.34**
Yield per plant (g)	47.62±2.16	57.19±2.65**
Actual yield per plot (kg)	9.25±0.36	10.98±0.41**
Actual yield increase over 93-11 (%)	–	18.70

Data are from plants in randomized complete block design with three replications under natural conditions in Hangzhou, China, in 2012. The planting density was 24.0 cm × 24.0cm, with one plant per hill. The area per plot was 12 m^2^. ^*^ and ^**^ indicate significant differences compared NIL-9311 to 93-11 at 0.05 and 0.01 levels, respectively. Values are means ± SD.

## DISCUSSION

### Japonica Allele LSCHL4 Better Coordinates Source–Sink Relationship and Increases Rice Yield

Grain yield has long been the main target of rice breeding, for which a large number of high-yield high-quality cultivars have been generated. According to a systematic analysis of representative rice cultivars developed over the past few decades, the increase in yield potential is mainly attributed to extended duration and increased leaf area for photosynthesis achieved by the improvement of plant type and the utilization of heterosis (Richards, 2000). Presently, how to further improve rice yield, optimize the late-stage relationship between photosynthetic level and nutrient use efficiency, and coordinate the source–sink relationship are the main directions for further exploring the yield potential.

For rice, the source and the sink have a mutual and coordinative relationship (Von and Farquhar, 1981; Zhu et al., 1997). Coordination of the source–sink relationship helps to improve the conversion efficiency of assimilates and thus increases the yield (Li et al., 2006); conversely, an excessively large source–sink ratio will reduce the degree of circulation in vascular bundles, negatively affecting assimilated transport and thus decreasing the yield. In recent years, multiple major genes related to the yield in rice have been cloned, and their relations with panicle type, thousand-grain weight, and the numbers of spikelets and branches per panicle have been confirmed. However, whether single genes can effectively improve the source–sink relationship in rice plants and be used directly for high-yield breeding of rice has rarely been reported.

In the present study, we introduced *LSCHL4*, an allele of *NAL1* from *japonica* rice cultivar, into the *indica* rice cultivar 93-11, which improved a cultivar of traits related to source–sink flow (e.g. flag leaf area, panicle length, the number of secondary branches and spikelets per panicle, and the number of vascular bundles per panicle exertion), ultimately increasing the yield of 93-11 in field cultivation (i.e. 18.55% increase in the near-isogenic line). According to the results, we propose that the gene *LSCHL4* allelic to *japonica* gene *NAL1* has pleiotropic effects, and thus can effectively improve the source–sink flow relationship and increase the yield in *indica* rice cultivar.

### Japonica Allele LSCHL4 Has Great Potential for High-Photosynthetic Efficiency Breeding

Crop yield is generally related to photosynthesis in leaves (Xu and Shen, 1994). Improvement of photosynthetic efficiency in leaf blade thus can effectively increase dry matter accumulation and yield in crops (Chen et al., 1995). For breeding the ideal plant type of rice, the major pathways presently accepted for improving leaf photosynthetic efficiency and grain yield are increasing light-receiving area, chlorophyll content, and photosynthesis time of leaves (Teng et al., 2004; Zhang et al., 2009; Jiang et al., 2010). However, how to achieve the consistency of regulating leaf morphology and increasing stay-green ability has long been a bottleneck problem that needs to be solved by plant breeders (Fu et al., 2009; Hörtensteiner, 2009). Several studies have reported that the *NAL1* allele is related to leaf morphological traits, photosynthetic efficiency, and panicle traits in rice, whereas the present study took the lead to employ a combination of molecular biology and conventional genetic breeding techniques to prove that *NAL1* allele from *japonica*, namely *LSCHL4*, once transformed through the transgenic or chromosome segment substitution method into rice cultivar with *indica* as the genetic background, can effectively improve the stay-green performance of leaves and increase leaf area, ultimately leading to a significant increase in rice yield. We believe that the near-isogenic line of *LSCHL4* in the genetic background of *indica* improves photosynthetic efficiency through coordination of leaf morphological traits and chlorophyll synthesis, which has great potential for use in breeding for high light-utilization efficiency.

### Pyramiding Heterotic Genes of Indica and Japonica Cultivars to Achieve New Breakthroughs of Yield in Super Rice

93-11 (Yangdao 6) is a popular Chinese super rice with *indica* as the genetic background, which has the advantages of high quality, multiple resistance, and high yield. 93-11 is a representative rice cultivar sequencing the genome framework. As mentioned above, how to achieve new breakthroughs of yield in super rice is a new challenge encountered in solving global food safety. Our results demonstrate that *NAL1* alleles from *indica* and *japonica* rice cultivars have substantially different functions, and that transduction of the *japonica NAL1* allele *LSCHL4* into the *indica* rice cultivar 93-11 significantly improves the yield of rice. This result lays a solid foundation for increasing the yield of super rice through modification of a single gene. Although *NAL1* allele from *japonica* rice cultivar is clearly an important gene that regulates leaf morphology and panicle traits, whether it has similar advantages in root development and nutrient uptake needs to be studied. Further exploration of and pyramiding more high-yield alleles resembling *NAL1* for super rice breeding by molecular design, in order to optimize plant type and the source–sink relationship, and further to achieve new breakthroughs in raising the yield in super rice and providing new ideas for solving the problem of global food safety are recommended.

### Effects of NAL1 Allele on Development of Rice Plant and Panicle Types in Different Populations

In recent years, substantial research has been conducted on the function of the *NAL1* allele. Qi et al. (2008) considered that *NAL1* affects the development of small vascular bundles through regulating polar auxin transport. Additionally, Chen et al. (2012), Takai et al. (2013), and Fujita et al. (2013) constructed RILs and chromosome segment substitution lines (CSSLs) using the American javanica cultivar D50 and the *indica* cultivar HB277, the *japonica* cultivar Koshihikari and the *indica* cultivar Takanari, and a new plant type of tropical *japonica* and the *indica* rice cultivar IR64, respectively; these authors further cloned *NAL1* allele including *qFLW4* related to leaf width, *GPS* related to photosynthetic efficiency, and *SPIKE* related to the number of branches per panicle. Between the *japonica* and *indica* subspecies, *NAL1* allele has relatively conservative differences in the coding region (mainly three single-base substitutions), which cause different effects in regulating the development of multiple traits related to plant type and panicle type.

The conclusions of previous research regarding *NAL1* allele have certain disparity with our results obtained with 93-11 and Nipponbare as the parents. According to Chen et al. (2012), *NAL1* allele in D50 can increase leaf width. The results of sequence comparison analysis show that the sequence of *NAL1* allele in D50 is identical with those in popular *indica* rice cultivars such as 93-11 and IR64. Thus, we presumed that *indica NAL1* allele could increase leaf width. On the contrary, our results showed that *japonica NAL1* allele from Nipponbare effectively increased leaf width and length, thus expanding leaf area. This result is consistent with previous findings by Wang et al. (2011).

According to the sequence data provided by Takai et al. (2013), we found that the *indica* cultivars 93-11 and Takanari share an identical coding sequence of *NAL1* allele, whereas the *japonica* cultivars Nipponbare and Koshihikari share a completely identical coding sequence of *NAL1* allele. We are in agreement that *NAL1* allele from *japonica* rice cultivars is capable of promoting leaf area increases, but the previously proposed source direction of an additive effect allele for chlorophyll content is opposite to our results. Takai et al. (2013) proposed that *NAL1* allele from the *indica* cultivar Koshihikari increases leaf chlorophyll content and thus deepens leaf color. Based on our results, we consider that the main cause for deepening of leaf color in NIL-9311 is related to the introduction of *NAL1* allele from the *japonica* cultivar Nipponbare. *NAL1* may be a key gene for the development of plant type and yield-related traits in rice. In a different genetic background, however, identical gene coding sequences may interact with other genes and result in differential gene expression and functions, ultimately affecting plant type and yield.

## METHODS

### Planting of Rice Materials and Survey of Agronomic Traits

The F_1_ population was constructed using the *indica* super rice cultivar 93-11 and the popular high-quality *japonica* cultivar Nipponbare, for which genome sequencing had been completed. Segregating individuals of the F_2_ generation were successively inbred through single seed descent for 12 generations. A total of 207 stable segregating individuals were obtained to form a RIL population and then planted at the experimental base of China National Rice Research Institute (N30°16’, E120°12’), Hangzhou, China. Rice seeds were sown on May 20 and transplanted on June 10, 2012. Each strain was planted in three rows, with six plants per row and three replicates for each treatment. For all plant materials, six individual plants were randomly selected from each stable strain at the initial heading stage and 20 d after heading, and six flag leaves per plant were randomly chosen for chlorophyll determination and leaf width measurement. SPAD values were determined in the upper, middle, and base sections of each flag leaf and the mean SPAD value of the leaf sections was taken as the phonotypical value of SPAD in individual plants. The chlorophyll content of flag leaves was calculated as follows: *Y* = 0.0996*X* − 0.152, where *X* is the SPAD value determined using a SPAD-502 leaf chlorophyll meter (Minolta, Japan), and *Y* is the actual leaf chlorophyll content (mg dm^−2^). Hereafter, leaf chlorophyll content is presented as SPAD value (A-JIA et al., 2008).

The maximal width of six flag leaves was measured using a scaled meter and the mean value of the repeated measurements was taken as the phonotypical value of flag leaf width in the individual plants (cm).

### Data Analysis and Linkage Map Construction

To analyze morphological traits and chlorophyll content in rice flag leaves, we mapped relevant QTLs in high-generation RILs constructed using the rice cultivars 93-11 and Nipponbare. Population distribution and correlation analysis were performed using the SAS 8.0 statistical software. A genetic linkage map was constructed using a total of 150 SSR and STS markers distributed evenly on all 12 chromosomes. The 1476.6-cM linkage map covering nearly the whole rice genome was used for QTL analysis. QTL mapping was conducted using the mapmaker/Exp3.0b software. QTLs in the DH population were detected using the software QGene 4.0 (Joehanes and Nelson, 2008) by the composite interval mapping (CIM) approach. Based on a permutation test (1000 permutations, *P* = 0.05) for all data sets, QTLs were determined using a threshold of 2.8. The genetic parameters, additive effects, and accounted variation of each QTL were also estimated. The relative contribution of a genetic component was calculated as the proportion of phenotypic variance explained by the component in the selected model (McCouch et al., 1997).

### Construction of Near-Isogenic Line

**Figure 8:**
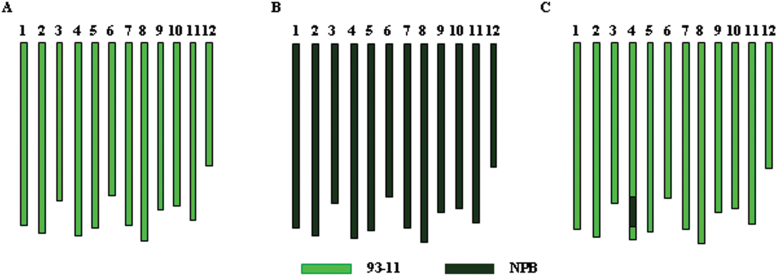
Graphical Genotypes of the Rice Cultivar Nipponbare, 93-11, and the Near-Isogenic Line *NIL-*9311. The numbers from 1 to 12 above boxes mean 12 chromosomes of indica 93-11 **(A)**, Nipponbare **(B)**, and NIL-9311 **(C)**, respectively. Light-green boxes in NIL-9311 mean the chromosome segment from the indica rice cultivar 93-11; dark-green boxes in NIL-9311 mean the chromosome segment from the japonica rice cultivar Nipponbare.

Based on the CSSLs of Nipponbare that had experienced four generations of successive inbreeding with 9311 as the recurrent parent, a CSSL of the donor parent Nipponbare (VD359) covering the QTL *LSCHL4* was screened out using the molecular markers STS4-5 and RM349 linked to *LSCHL4*. VD359 was backcrossed with 9311 for two generations followed by inbreeding. Additionally, marker-assisted selection was performed using 120 pairs of molecular markers covering the sequence of the whole genome. A near-isogenic line (*NIL*-9311) derived from single segment substitution lines (SSSLs) of Nipponbare and carrying *LSCHL4* was screened out, and then used for the survey of leaf morphological traits and yield-related traits (Figure 8).

### Fine Mapping of qLSCHL4

To isolate the QTL *qLSCHL4*, the near-isogenic line NIL-9311 was hybridized with 9311 to generate BC_7_F_1_, which was then used to generate a BC_7_F_2_ population via inbreeding. The SPAD value of all plants was measured at the heading stage. Additionally, 10 STS primer pairs with polymorphism and six SSR primer pairs (Table 3) were designed and synthesized for fine positioning and map-based cloning of the QTL *qLSCHL4*.

**Table 3:** Molecular Markers on Chromosome 4 for Map-Based Cloning and Identification of *qLSCHL4*

Marker	F	R	cM
RM273	GAAGCCGTCGTGAAGTTACC	GTTTCCTACCTGATCGCGAC	94.4
T2957–2	GATATCGCTAGCGGATTCAGAG	TCCGTAGTGTAGTGATATGGTGA	97.7
T6684–1	ATACACCTTTTATGGCATGG	AGTTGAAGGGCTCTCTCTCT	102.7–107.4
T1610–1	CATATACTCCCTTCATCCCA	CATTTTAACCGAGCATCAAT	102.7–107.4
T2950–1	TTCTTCTGATGGGCCGTCTATG	ATCGGCTTTCCTTAGTCTCACG	102.7–107.4
T2950–2	GCCGTCCGTTTTGTCGTG	CGAGGAACACCAACTACACTGT	102.7–107.4
T2950–3	AGCTAGAGAAAATAAGGGCGCT	ACGCAGGAATCAGATGAGAGAG	102.7–107.4
T2950–4	TAGTGCCGCATCTTCAAATTGC	ACGATAAAGCAAACCCTAAACCC	102.7–107.4
T2950–5	GCTCTACTACGAGGGCATGATG	CCGTCATGCCCTCAGATGA	102.7–107.4
T6648–1	ACACACCAAATGGAACTACC	TGCAAATTGAAGTGAGACTG	102.7–107.4
RM3839	AATGGGACCAGAAAGCACAC	AAAAAGAGCATGGGGGCTAC	72.8
RM303	GCATGGCCAAATATTAAAGG	GGTTGGAAATAGAAGTTCGGT	87.1
T6455–1	GGGACCTTGAGTGAACGG	GGATTTTGGGCTCTGCC	102.1
RM349	TTGCCATTCGCGTGGAGGCG	GTCCATCATCCCTATGGTCG	112
RM348	CCGCTACTAATAGCAGAGAG	GGAGCTTTGTTCTTGCGAAC	113.2
RM1113	GGGCGCATGTGTATTTCTTC	TGGGGAAAAACCACAAGCC	123.8
RT-52479	ACATTGGGGATGTCAAGGTTAT	CATCACAGTCCCAGTTGTGTG	LOC_Os04g52479
RT-52500	ACTCCCAACAGCCTTCGAGA	ACATAGGAGGAAGCTCGGC	LOC_Os04g52500
RT-52504	GACTACGGCGGGTATCAGCA	TCAGAAGCACATGTCGAGC	LOC_Os04g52504

### Microscopic and Histologic Examinations

According to Hu et al. (2010), flag leaf samples were collected from parental cultivars Nipponbare and 93-11 and the near-isogenic line NIL-9311 at the heading stage. The leaves were fixed in FAA (containing 50% anhydrous ethanol, 0.9mol L glacial acetic acid, and 3.7% formaldehyde) at 4°C for over 16h, followed by dehydration using gradient ethanol, clearing with dimethylbenzene, and embedding with paraffin. The samples were sectioned into 10-μm slices, cross-stained with 1% sarranine and 1% Fast Green reagent, and then examined under a 90i light microscope (Nikon, Japan).

### Real-Time Quantitative Polymerase Chain Reaction (qRT–PCR) Assay

Differential expression in the parental cultivar 93-11 and the near-isogenic line NIL-9311 of three candidate genes contained in the mapped gene interval was detected via real-time qPCR assay. Total RNA was extracted from flag leaves at heading and maturity stages using a RNeasy plant mini kit (Qiagen, Hilden, German). The RNA extract was purified using DNase I from TaKaRa and then reverse-transcribed into cDNA using a ReverTra Ace-2 kit (Toyobo, Osaka, Japan). Gene-specific primers were designed using the online tool Primer3.0 (http://primer3.ut.ee) according to the full-length cDNA sequences of three candidate genes (LOC_Os04g52479, LOC_Os04g52500, and LOC_Os04g52504; http://rice.plantbiology.msu.edu/cgi-bin/gbrowse/rice/#search). Relative expression levels of the three were assayed on a real-time PCR system (ABI 7900, USA).

### Vector Construction and Transformation

The cDNA fragment containing the entire coding region *NAL1* gene was amplified using primers containing the *Sma*I and *Sal*I sites (5′-*CCCGGG*ATGAAGCCTTCGGACGATAA-3′ and 5′-*GTCGAC*TCATTTCTCCAGGTCAAGGCTT-3’) obtained by reverse-transcription PCR (RT–PCR). The digested fragment (1.749kb) was inserted into a KAN-resistant binary vector pCAMBIA2300-Actin1-ocs at the *Sma*I and *Sal*I sites, containing the cassettes of the rice actin (Act1) promoter and the nos terminator. This fragment was introduced into the Nipponbare (*japonica*) plant by *Agrobacterium tumefaciens*-mediated transformation for the overexpression test (Qi et al., 2008). The obtained transgenic plants and the wild-type parent Nipponbare were planted in a transgenic garden at the experimental base of the Chinese Rice Research Institute. Survey of agronomic traits was carried out as described above.

### Field Plot Experiment and Leaf Trait Survey

For field plot experiments, the donor parental cultivar Nipponbare, the recurrent parent cultivar 9311, and the near-isogenic lines NIL-9311 were planted in an experimental base of the China National Rice Research Institute, Hangzhou, China. The cultivars were seeded on May 20 and transplanted on June 10, 2012. The planting area of each plot was 12 m^2^ (1.2 m × 10.0 m), with six plants per row and row spacing at 24 cm × 24cm. Leaf traits were surveyed according to Gao et al. (2013) and Wang et al. (2012), including the SPAD value and width of flag leaves at the heading and maturity stages, the number of effective panicles at the heading stage, and panicle traits after harvesting (30 d after heading, including the numbers of primary and secondary branches per panicle, the number of effective panicles, the numbers of spikelets and grains per panicle, seed-setting rate, and thousand-grain weight). The measurement of all traits followed the Standard Evaluation System for Rice (www.knowledgebank.irri.org/ses/).

## FUNDING

This work was supported by grants from the National Natural Science Foundation of China (31221004 and 91335105) and the Provincial Science Fund for Distinguished Young Scholars of Zhejiang (R3100100). The funders had no role in study design, data collection and analysis, decision to publish, or preparation of the manuscript. No conflict of interest declared.
